# Left Atrial Appendage Closure: A Single-Center Experience in a Population With a High Prevalence of End-Stage Renal Disease

**DOI:** 10.7759/cureus.69286

**Published:** 2024-09-12

**Authors:** Luis A Areiza, Juan F Rodriguez, David Rodriguez

**Affiliations:** 1 Interventional Cardiology Department, Hospital Universitario Mayor Méderi, Bogotá, D.C., COL

**Keywords:** stroke, hemorrhage, anticoagulants, atrial fibrillation, atrial appendage

## Abstract

Background

Left atrial appendage closure (LAAC) has emerged as an alternative approach for mitigating thrombotic risk in nonvalvular atrial fibrillation patients. However, existing registries often lack representation of the Hispanic population, motivating this study to elucidate the demographic, clinical, and procedural characteristics, specifically among Hispanic patients undergoing this procedure.

Methods

Adult patients who underwent percutaneous LAAC between June 2017 and July 2022 at a high-complexity hospital in Bogotá, COL, were included. Baseline and procedural characteristics are reported. For patients with available follow-up data, major bleeding, thromboembolic events, and cardiovascular mortality were assessed. A subgroup analysis was conducted for patients with end-stage renal disease on dialysis.

Results

We included 33 patients. Follow-up data were available for 27 patients, with a mean follow-up period of 12.4 months. The median age of the cohort was 70 years (SD 9), with 58% being women. The median CHADS2 and HAS-BLED scores were 3 points (IQR 2 to 4) and 4 points (IQR 3 to 4), respectively. The 90-day cardiovascular mortality rate was 3.7%, whereas cardioembolic episodes and major bleeding events were reported at rates of 10.8 and 14.4 per 100 patient years, respectively. The long-term outcomes of patients on dialysis were comparable to those of nondialysis patients.

Conclusions

Our study reinforces existing evidence supporting the safety of LAAC, particularly in a multimorbid patient population with elevated bleeding and thrombotic risks. In this high-risk cohort, LAAC emerges as a feasible alternative for reducing thromboembolic risk. Notably, patients on dialysis demonstrated comparable long-term outcomes, suggesting the procedure's viability in this subgroup as well.

## Introduction

Atrial fibrillation is the most common cardiac arrhythmia in adults [1]. Chaotic and inefficient atrial contraction generates a prothrombotic environment, increasing the risk of thromboembolic events. Anticoagulant therapy is the cornerstone in the management of atrial fibrillation. Predictive scores such as the CHADS2 score are used to determine the need for this therapy [2]. Despite the significant reduction in thromboembolic risk from anticoagulants, these drugs also carry a hemorrhagic risk that might be prohibitive for some patients. There is no consistent evidence to support the use of these drugs in select groups of patients, such as those with end-stage chronic renal disease [3].

Up to 91% of atrial thrombi are found in the left atrial appendage (LAA) [4], which makes percutaneous left atrial appendage closure (LAAC) an alternative treatment. The safety of the procedure and its noninferiority in preventing thrombotic events have been demonstrated in various studies. However, existing registries often lack representation of the Hispanic population, motivating this study to elucidate the demographic, clinical, and procedural characteristics, specifically among Hispanic patients undergoing this procedure. This study aimed to describe the demographic and clinical variables, procedure characteristics, and outcomes of patients who underwent percutaneous LAAC at a high-complexity hospital in Bogota, COL.

## Materials and methods

Study design

This was an observational retrospective study. Adult patients who underwent percutaneous LAAC between June 2017 and July 2022 at a high-complexity hospital in Bogotá, COL, were included. Patient demographics and clinical and technical data related to the procedure and outcomes were collected from electronic clinical records. This study received approval from the Research Ethics Committee of the Universidad del Rosario Life Science Room (approval no. DVO005 2472-CV1806), and informed consent was waived because of the minimal risk to participants.

Left atrial appendage closure and postprocedural management

Patients referred by primary care physicians were evaluated by a multidisciplinary board to determine eligibility for LAAC. Patients with contraindications to anticoagulation, a high risk of hemorrhage, nonadherence to therapy, or persistent thromboembolic events despite anticoagulation were assessed.

Procedure planning included prior transesophageal echocardiography (TEE) or cardiac CT. The procedure was performed under general anesthesia or conscious sedation at the discretion of the anesthesiology and interventional cardiology teams. Watchman^TM^ (Boston Scientific, Marlborough, MA, USA) and Amplatzer/Amulet^TM^ (Abbott, Chicago, IL, USA) LAAC devices were used, with device selection and sizing determined by the interventional cardiologist on the basis of morphological characteristics and expertise. Fluoroscopy and TEE were used for intraprocedural guidance and to confirm successful device implantation. Dual antiplatelet therapy was formulated for three to six months after LAAC, followed by indefinite low-dose aspirin.

Endpoints and definitions

Procedure-related complications and in-hospital mortality were evaluated for all included patients. For patients with available follow-up data, 90-day cardiovascular mortality, all-cause mortality, and the occurrence of hemorrhagic or cardioembolic events during follow-up were assessed.

Major bleeding was defined according to the criteria used for the STEEPLE trial [5]. The left ventricular ejection fraction was calculated via transthoracic echocardiogram with the biplane Simpson method, the left atrial volume index was calculated from TEE measurements, and the LAA morphology was classified by two independent cardiologists using TEE and fluoroscopy.

Statistical analysis

For the descriptive analysis of the variables, we applied the Shapiro-Wilk test to assess the normality of the distributions. Means and standard deviations (SDs) are reported for normally distributed variables, whereas medians and interquartile ranges (IQRs) are reported for non-normally distributed variables. Categorical variables are expressed as absolute and relative frequencies.

An exploratory univariate logistic regression analysis was conducted to identify potential predictors for the occurrence of a composite endpoint of mortality, hemorrhage, or cardioembolic events during follow-up. As none of the variables included in the univariate model were significant, no multivariate analysis was performed. The association between LAA morphology and mean fluoroscopy time was assessed via ANOVA, followed by Tukey's honestly significant difference (HSD) post hoc test.

Finally, a subgroup analysis comparing the nondialysis and dialysis populations was performed. The occurrence of the endpoints was compared with odds ratios (ORs), and 95% confidence intervals (CIs) were reported. The level of significance was set at a p-value ≤ 0.05. All the statistical analyses were performed using R statistical software (version 4.2.3 (R Foundation for Statistical Computing, Vienna, AUT).

## Results

Baseline and procedure characteristics

A total of 33 patients were included in the study. Six patients did not have available follow-up data and were excluded from the long-term endpoint analysis. Among the other 27 patients, the mean follow-up was 12.4 months. The patients’ baseline characteristics are summarized in Table 1. The mean age was 70 (±9) years, and 19 (42%) patients were females. A history of renal replacement therapy was present in 17 (52%) patients. Stroke and major bleeding histories were present in 17 (52%) and 27 (82%) patients, respectively. The median CHADS2 score was 3 (IQR: 2 to 4) points and the median HAS-BLED score was 4 (IQR 3 to 4) points. The median ejection fraction (EF) was 55% (IQR 42% to 59%), with 64% of the patients presenting a normal EF, 15% presenting a mildly reduced EF, and 21% presenting a reduced EF. The mean left atrial volume index was 53.2 (±19.5) ml/m2.

**Table 1:** Baseline characteristics Total n = 33 CKD: Chronic kidney disease, EF: Ejection fraction, HFmEF: Heart failure with mid-range ejection fraction, HFrEF: Heart failure with reduced ejection fraction, DOACs: Direct oral anticoagulants, SD: Standard deviation, IQR: Interquartile range

Characteristics	Values: N (%)
Age (in years)	69.9 (SD: ±9.1)
Sex	
Female	19 (57.6%)
Male	14 (42.4%)
CKD stage 3-4	5 (15.2%)
Renal replacement therapy	17 (51.5%)
Hypertension	27 (81.8%)
Diabetes mellitus	11 (33.3%)
Stroke history	17 (51.5%)
Major bleeding history	27 (81.8%)
CHADS2 score, median (IQR)	3 (IQR: 2 to 4)
1	6 (18.2%)
2	9 (27.3%)
3	9 (27.3%)
4	6 (18.2%)
5	2 (6.1%)
6	1 (3%)
HAS-BLED score, median (IQR)	4 (IQR: 3-4)
1	1 (3%)
2	4 (12.1%)
3	4 (12.1%)
4	17 (51.5%)
5	7 (21.2%)
Left ventricular EF, % (IQR)	55% (IQR: 42-59)
Normal EF	21 (63.6%)
HFmEF	5 (15.2%)
HFrEF	7 (21.2%)
Left atrial volume index, ml/m^2^	53.2 (SD: ±19.5)
Primary indication for the procedure	
High risk of hemorrhage and previous major event	8 (24.2%)
Contraindications for oral anticoagulation	4 (12.1%)
Failure of oral anticoagulation	21 (63.6%)
Preprocedure anticoagulation	
DOACs	7 (21.2%)
Vitamin K antagonist	9 (27.3%)
Heparin	12 (36.4%)
No anticoagulation	5 (15.2%)

The procedure characteristics are presented in Table 2. The mean fluoroscopy time was 1465 minutes (1082 to 2011 minutes). The median device size was 25 mm (IQR: 24 mm to 31 mm), and 27 (81.8%) patients received a Watchman device for LAAC. The most common LAA morphology was the windsock morphology (n = 18; 55%), followed by the chicken wing morphology (n = 8; 24%) and the cauliflower morphology (n = 7; 21%).

**Table 2:** Procedure characteristics Total n = 33 IQR: Interquartile range

Characteristics	Values
Fluoroscopy time (minutes)	1465 (IQR: 1082-2011)
Device size (mm)	25 (IQR: 24-31)
Watchman device	27 (81.8%)
Amulet device	6 (18.2%)
LAA morphology, n (%)	
Windsock	18 (54.6%)
Chicken wing	8 (24.2%)
Cauliflower	7 (21.2%)

Outcomes

One procedure-related complication was documented, and one patient died before discharge due to a noncardiovascular cause, with each representing 3%. For patients with available follow-up data (n = 27), the 90-day cardiovascular mortality rate was 3.7%. Seven patients died during follow-up, three experienced thromboembolic events, and four experienced major bleeding events (Table 3). The incidence density was 10.8 thromboembolic episodes per 100 patient years and 14.4 major bleeding events per 100 patient years. An exploratory logistic regression analysis could not identify specific predictors for the occurrence of the composite endpoint (Table 4).

**Table 3:** Outcomes SD: Standard deviation

Outcome	Values: N (%)
Acute outcomes	N = 33
Complications	1 (3%)
In-hospital mortality	1 (3%)
Long-term outcomes	N=27
Mean follow-up (months)	12.4 (SD: ±14.2)
90-days cardiovascular mortality	1 (3.7%)
All-cause mortality during follow-up	7 (25.9%)
New thromboembolic events	3 (11.1%)
New major bleeding	4 (14.8%)

**Table 4:** Logistic regression analysis for variables predicting the composite endpoint CKD: Chronic kidney disease

Univariate analysis	OR (95% CI)	p-value
Age	1.07 (0.98-1.18)	0.13
Sex (male)	0.38 (0.08-1.86)	0.22
CKD stage 3 or 4	1.52 (0.3-7.69)	0.6
Dialysis	2.1 (0.46-9.58)	0.33
Hypertension	1 (0.15-6.79)	1
Diabetes	4 (0.84-19.84)	0.08
Stroke history	0.4 (0.09-1.82)	0.22
Major bleeding history	2.9 (0.29-30.27)	0.36
HAS-BLED score	1.26 (0.59-2.71)	0.56
CHADS2 score	1.25 (0.7-2.22)	0.45
Left ventricular ejection fraction	0.99 (0.94-1.05)	0.79
Left atrial volume index	1.03 (0.99-1.08)	0.15

Impact of LAA morphology on fluoroscopy time

**Figure 1:**
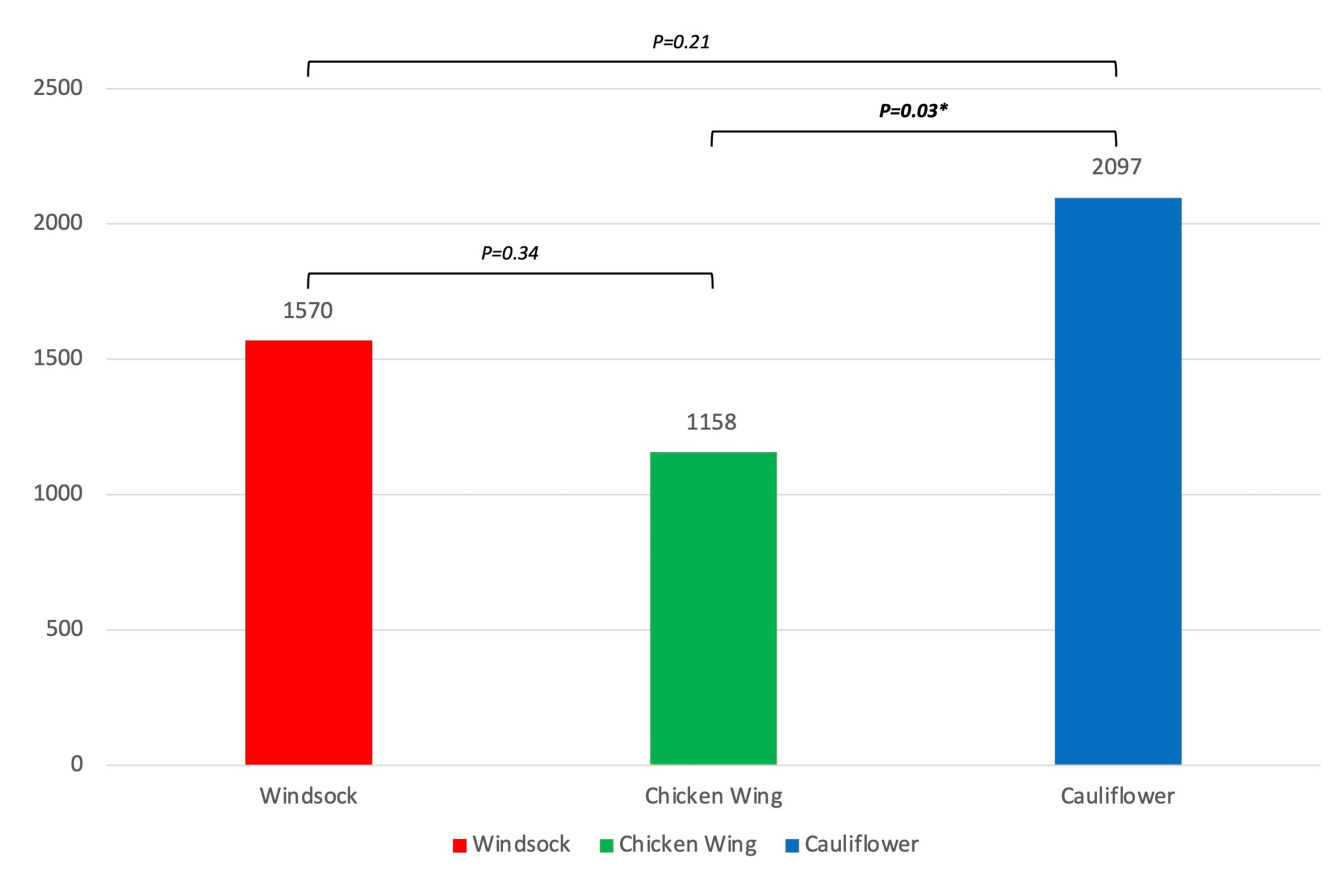
Fluoroscopy time and LAA morphology The fluoroscopy time in the cauliflower group was longer than in the chicken wing group (mean difference: 939 sec; 95% CI: 70 to 1808 sec; p = 0.03). LAA: Left atrial appendage

The mean fluoroscopy time was compared among the three different types of LAA morphologies present in our population (Figure 1). Chicken wing morphology required a shorter fluoroscopy time (1158 sec), followed by windsock (1570 sec) and cauliflower (2097 sec). A significant difference between chicken wing morphology and cauliflower morphology was documented (mean difference: 939 sec; 95% CI: 70 to1808 sec; p = 0.03).

Subgroup analysis for end-stage renal disease

Baseline characteristics stratified by history of dialysis are presented in Appendix A. The prevalence of diabetes was greater in the dialysis population; the other baseline characteristics had a balanced distribution. Acute complications, in-hospital mortality, and 90-day mortality were not compared because of the small number of events. The follow-up period was comparable between the two groups. There was a trend toward higher all-cause mortality in the dialysis population, but the difference was not statistically significant (OR: 8.3; CI 95%: 0.8-446; p = 0.08). No differences were observed in new thromboembolic or major bleeding events (Table 5).

**Table 5:** Subgroup analysis and outcomes in nondialysis vs. dialysis SD: Standard deviation

Outcome	Nondialysis	Dialysis	OR (95% CI)	p-value
Acute outcomes	N=16	N=17		
Complications	1 (6.3%)	0 (0%)	NA	NA
In-hospital mortality	0 (0%)	1 (5.9%)	NA	NA
Long-term outcomes	N= 13	N=14		
Mean follow-up	15 (SD: ±15.6)	9.9 (SD: ±12.8)		0.37
90-days cardiovascular mortality	0 (0%)	1 (7.1%)	NA	NA
All-cause mortality during follow-up	1 (7.7%)	6 (42.9%)	8.3 (0.8-446)	0.08
New thromboembolic events	2 (15.4%)	1 (7.1%)	0.44 (0.01-9.5)	0.6
New major bleeding	2 (15.4%)	2 (14.3%)	0.92 (0.06-14.8)	1

## Discussion

Main results

The complication and in-hospital mortality rates were comparable to those reported in previous studies [6]. Baseline characteristics, especially the significant proportion of patients on dialysis, may have impacted the mortality rate. Thromboembolic and major bleeding event rates were higher than those previously reported [7], which may be explained by the demographic and clinical characteristics of our population. In our study, the cauliflower morphology appeared to be particularly challenging and was associated with significantly longer fluoroscopy times. Patients on dialysis tended to have higher mortality, although the difference was not significant compared with nondialysis patients.

Previously reported safety and effectiveness of LAAC

For the Watchman device, the PROTECT AF study included 707 randomized patients treated with vitamin K antagonist anticoagulation and percutaneous LAA closure via the Watchman device. For compound events such as stroke, cardiovascular death, or systemic embolism, noninferiority criteria were met after 1065 patient years of follow-up [8]. In terms of complications related to the procedure, the EWOLUTION register reports a 2.8% risk [6].

For the Amulet device, the risk of adverse events related to the procedure has been estimated at 3.2% [9]. In terms of thromboembolic prevention, records of stroke incidence at one year are available, with annual rates of 2.3% in a population with a calculated annual stroke risk of 5.6% according to the CHA2DS2-VASc score [10].

Impact of LAA morphology

The traditional morphological classifications of the LAA are chicken wing, cactus, windsock, and cauliflower. Lobule localization, number, and predominance are the main differences between the groups. The chicken wing and windsock morphologies involve a dominant lobule, but the former has a middle or proximal fold. The cactus morphology includes a central dominant lobule with a variable number of secondary lobules. Finally, the cauliflower morphology is a short appendage with multiple, nondominant lobules [11].

Transesophageal echocardiogram, cardiac angiotomography, and fluoroscopy are available techniques for morphological characterization of the LAA. These techniques also allow the documentation of intracavitary thrombi that contraindicate percutaneous closure with a device. In our study, morphological assessment was performed via TEE and fluoroscopy. The most common morphology was chicken windg. There was a significant difference in fluoroscopy time between chicken wing and cauliflower, which might be explained by the procedural and technical implications of the morphological variables.

Complications

Cardiac tamponade is an early complication that usually occurs within the first 24 hours. The use of TEE or intracardiac ultrasound as a visual aid during the procedure is recommended to reduce the risk of this complication [12]. The incidence of cardiac tamponade has decreased as operators have increased their expertise. For the Watchman device, the incidence has been estimated to be 1.3% [13].

The incidence of device thrombosis is 3.9% [14]. Experimental studies of the Watchman device have shown that the epithelization time is between 30 and 90 days [15]. The coagulation cascade reaches its maximum activity seven days after implantation, as measured by biomarkers [16]. Less frequent complications include stroke and device embolization. Preprocedure evaluation to rule out preformed thrombi and correct estimation of the device size is essential to reduce the probability of these events [17].

In our study, 3% of the patients presented complications related to the procedure. The only reported event was thrombosis of the interauricular septum, which was not associated with cardioembolic events. These results are consistent with the reported complication rates. Improvements in the techniques and devices used can further reduce the current complication rates.

Left atrial appendage closure in chronic kidney disease (CKD) and end-stage CKD

Patients with CKD represent a challenging population for this intervention. The use of contrast media during the procedure in this group can increase the risk of contrast-induced nephropathy and approaches avoiding the use of contrast agents have shown promising results [18]. As anticoagulation is a challenge for patients on dialysis, LAAC emerges as an alternative, although evidence in this population is limited. Comparable outcomes have been reported in patients with end-stage renal disease [19, 20]. A national registry study found that for patients with stage 5 CKD, the 90-day stroke and bleeding rates were 1.7% and 7.2%, respectively. Multivariate analysis indicated that these rates did not differ significantly when compared to patients without CKD [21]. Further studies are needed to define the scope of LAAC in this population. In our study, patients on dialysis demonstrated similar long-term outcomes compared to the nondialysis population.

Challenges in postprocedural antiplatelet therapy and anticoagulation management

Postprocedure antithrombotic management is a highly debated topic. Given that most patients undergoing this procedure have a very high risk of bleeding or, in some cases, even contraindications to anticoagulant use, defining a pharmacological strategy to reduce the thrombotic risk associated with the device before achieving device epithelialization is a challenge.

Pivotal studies have used vitamin K antagonists and aspirin. Nonrandomized clinical trials have compared dual antiaggregation therapy with vitamin K antagonist use alone. Sondergaard et al. performed a matched pair analysis of 1527 patients receiving oral anticoagulation therapy and 509 patients receiving dual antiplatelet therapy, and no significant differences were found between the two groups in terms of thromboembolic events or bleeding at the six-month follow-up. The risk of device thrombosis was greater in the double antiaggregation group (p = 0.014) [22].

Fractionated heparin administration is recommended during the procedure with a goal of 250 sec activated clotting time (ACT). For the Watchman device, lifelong treatment with low-dose aspirin and a vitamin K antagonist for 45 days followed by clopidogrel for six months is recommended. Oral anticoagulation is not recommended for patients with a very high bleeding risk who are receiving only six months of P2Y12 inhibitor therapy and lifelong low-dose aspirin. For the Amulet device, one to six months of low-dose aspirin and clopidogrel are recommended. There is not enough evidence to recommend the use of nonvitamin K antagonists [17].

Limitations 

This study has several limitations. First, it is an observational retrospective study, which implies a high risk of selection bias. Second, the sample size is small, and a significant number of patients were lost to follow-up. Third, no control arm was included to compare this approach with an oral anticoagulation strategy. Finally, some data, such as postprocedural echocardiographic measurements, were not available for collection and therefore not reported.

## Conclusions

Our study reinforces existing evidence supporting the safety of LAAC, particularly in a multimorbid patient population with elevated bleeding and thrombotic risks. In this high-risk cohort, LAAC emerges as a feasible alternative for reducing thromboembolic risk. Notably, patients on dialysis demonstrated comparable longterm outcomes, suggesting the procedure's viability in this subgroup as well. Prospective studies and larger registries are still necessary to improve the evidence supporting this therapy in Hispanic patients.

## Appendices

Appendix A

Table 6 features baseline characteristics of the patients stratified by history of dialysis. 

**Table 6:** Baseline characteristics disaggregated by history of dialysis EF: Ejection fraction, HFmEF: Heart failure with mid-range ejection fraction, HFrEF: Heart failure with reduced ejection fraction, DOACs: Direct oral anticoagulants

Variable	Non-dialysis (n = 16)	Dialysis (n = 17)	p-value
Age (years)	73.1 (±8.8)	66.9 (±8.4)	0.05
Male	7 (44%)	7 (41%)	0.88
Hypertension	12 (75%)	15 (88%)	0.33
Diabetes mellitus	2 (13%)	9 (53%)	0.03*
Stroke history	11 (69%)	6 (35%)	0.06
Major bleeding history	13 (81%)	14 (82%)	0.94
CHADS2 score	2.5 (2-4)	3 (2-3)	0.63
HAS-BLED score	4 (2.75-4)	4 (4-4)	0.31
Left ventricular EF, %	54.5 (40.3-59.3)	55 (47-59)	0.56
Normal EF	9 (56%)	12 (71%)	0.39
HFmEF	3 (19%)	2 (12%)	0.66
HFrEF	4 (25%)	3 (18%)	0.65
Left atrial volume index, ml/m^2^	49 (±20)	57.1 (±18.7)	0.25
Preprocedure anticoagulation			
DOACs	7 (44%)	0	NA
Vitamin K antagonist	3 (19%)	6 (35%)	0.44
Heparin	3 (19%)	9 (53%)	0.07
No anticoagulation	3 (19%)	2 (12%)	0.66
